# Influencing factors of suicidal ideation in lung cancer patients in Midland China: A mixed-method study

**DOI:** 10.3389/fpsyt.2023.1072371

**Published:** 2023-04-27

**Authors:** Ting Yu, Deying Hu, Yan Jiang, Cong Wang, Shanshan Liu

**Affiliations:** ^1^Evidence-based Nursing Center, West China Hospital, Sichuan University/West China School of Nursing, Sichuan University, Chengdu, China; ^2^Department of Nursing, Union Hospital, Tongji Medical College, Huazhong University of Science and Technology, Wuhan, China; ^3^Department of Nursing, West China Hospital, Sichuan University/West China School of Nursing, Sichuan University, Chengdu, China

**Keywords:** suicidal ideation, influencing factors, lung cancer, inventory survey, qualitative research

## Abstract

**Introduction:**

The suicide risk of lung cancer patients is higher than that of patients with other cancers. However, as China is a large country for lung cancer, there are no relevant reports on lung cancer suicides. This study aimed to investigate the prevalence of suicidal ideation and explore its influencing factors in lung cancer patients.

**Methods:**

In this cross-sectional study, between July to November 2019, 366 lung cancer patients from the oncology department of a general hospital in Wuhan were chosen as participants. Of these, eight with lung cancer and suicidal ideation were selected for in-depth interviews.

**Results:**

A total of 22.68% of lung cancer patients reported suicidal ideation. Sex, cancer stage, number of uncomfortable symptoms, and satisfaction with treatment were independently associated with suicidal ideation. This qualitative study found that the experience of suicidal ideation in lung cancer patients includes physiological (heavy burden of symptoms),psychological (bad mood, thwarted belongingness, perceived burdensomeness, and stigma), and social factors (high economic pressure and negative life events).

**Discussion:**

These findings suggest that the incidence of suicidal ideation in lung cancer patients is higher than that of other cancers and is affected by many factors. Therefore, there should be routine screening and assessment of suicidal ideation among lung cancer patients, and related mental health and suicide prevention education should be provided.

## Introduction

Lung cancer is associated with a malignant tumor and has the highest mortality rate in the world, ranking first and second in the incidence of male and female patients with cancer, respectively (1). Factors such as high lethality, poor prognosis, heavy symptom burden, and great economic pressure can easily induce psychological and social pressure on patients and break their psychological defense line, particularly in the context of the coronavirus disease (COVID-19) pandemic (2). A previous study showed that 92.8% of patients with lung cancer reported depression and anxiety (3). These are high-risk factors for suicide. A survey showed that patients with lung cancer have the highest risk of suicide, with a standard mortality ratio (SMR) of 4.17 (4). Additionally, several studies, including those from Canada, Austria, and Sweden, found that the suicide risk in patients with lung cancer was higher than that of patients with other cancers (5, 6). Lung cancer is associated with a malignant tumor and has the highest incidence and fatality rate in China (7). Therefore, suicide in lung cancer patients is a serious issue that needs to be paid attention to so that their safety can be ensured.

Suicidal ideation means that an individual expresses their wish to end their own life but does not take any actual action (8). The first step to better understand suicidal behavior is to identify and understand the present situation and influencing factors of suicidal ideation (9). Scholars have conducted related studies on patients with other cancers, such as gynecological (10) and stomach cancer (11), and have found that patients with low family function, poor self-conscious health, advanced tumor stage, and symptoms of clinical discomfort and depression, have higher suicidal ideation. Scholars have conducted many studies on symptom burden, economic pressure, and depression in patients with lung cancer. However, few studies have examined the factors influencing suicidal ideation, such as the lack of understanding of the sociality and particularity of this problem, and insufficient understanding of the complexity of suicidal ideation-related experiences and behaviors. Owing to differences in social and cultural backgrounds and the development of lung cancer, clinical symptoms and psychological conditions differ from those of other cancers, and the factors affecting suicidal ideation may differ as well. Therefore, urgent analysis of the factors affecting suicidal ideation in patients with lung cancer is needed.

It is difficult to fully understand the reasons for suicidal ideation using a single quantitative or qualitative study. In China, suicide is a sensitive social issue, and patients tend to hide their thoughts because of the influence of traditional culture. However, qualitative studies can describe, review, and analyze personal experiences through in-depth interviews to uncover important information.

Therefore, first (Part 1), this study adopted a cross-sectional survey to understand the current situation regarding suicidal ideation in lung cancer patients and to analyze their demographic and disease characteristics. Second (Part 2), based on a quantitative study, lung cancer patients with suicidal ideation were purposefully selected for in-depth interviews to obtain a deeper understanding of its influencing factors. Thus, it was hypothesized that lung cancer patients have a high incidence of suicidal ideation and are affected by physiological, psychological, and social factors. Therefore, the factors influencing suicidal ideation in lung cancer patients can be understood, and exploring the process of suicidal ideation helps us understand the occurrence of suicidal behavior.

## Methods

### Part 1: Quantitative component of the study

#### Design and participants

A questionnaire-based cross-sectional survey was conducted. Using a convenience sampling questionnaire, between July to November 2019, lung cancer patients from the oncology department of a general hospital in Wuhan were chosen as participants of the study. A total of 380 participants were recruited, and 366 of the questionnaires completed by the participants were valid. The incomplete questionnaires of four patients were excluded and discontinued because some patients were sensitive to the idea of death, suicide, and hopelessness. The questionnaires of three patients were excluded due to obvious logical inconsistencies. Additionally, seven patients were excluded because they had a history of mental illness or were taking related medications. The inclusion criteria were as follows: (a) age above 18 years; (b) primary diagnosis of lung cancer by histological examination; (c) capacity to be independently conscious and understand the contents of the study; and (d) knowledge about the diagnosis of cancer, voluntary participation, and completion of informed consent. The exclusion criteria were as follows: (a) patients with multiple cancers; (b) history of mental illness; (c) patients with antipsychotic drug prescription; and (d) incomplete questionnaires or questionnaires with inconsistent answers. This study was approved by the ethics committee of the relevant institution.

According to the calculation formula of the sample size of the cross-sectional survey (12), the incidence of suicidal ideation in patients with other cancers in China was also referred to (10), and a 20% loss to follow-up rate was considered. At least 284 patients were required for this study.

#### Measures

##### General information questionnaire

Based on a literature review and expert consultation, a questionnaire for general information was developed and included two parts: demographic characteristics, such as sex, age, marital status, educational attainment, and religion; and disease-related factors, such as lung cancer type, stage, number of hospitalizations, and satisfaction with treatment.

##### Patient health questionnaire-9

Patient Health Questionnaire-9 (PHQ-9) is a single-dimensional scale adopted from Kroenke et al. (13). There are nine items in the questionnaire, with scores ranging from 0 to 3. Item 9 asks “How often have you thought that you would be better off dead or hurting yourself in some way during the past two weeks?” For this item, a lower score (>0) indicates positive suicidal ideation, and a higher score indicates more serious suicidal ideation. The Chinese version of PHQ-9 has been applied to screen suicidal ideation in cancer patients in hospitals and has been widely recognized (14). Cronbach’s alpha in this study was 0.89.

#### Data collection

Data were collected using self-reported questionnaires and patients’ medical records. After obtaining the consent of the relevant departments, patients who met the standards were instructed to complete the questionnaire independently. Those who could not read and complete the questionnaire independently could be assisted by a researcher with no suggestive or induced language during the entire process. To ensure the quality of the questionnaire, it was completed on the day of the survey. Therefore, the investigation was conducted when the physical condition of the patients or time permitted it, and each patient was informed that the investigation would last for 30 to 45 min. The participants had the right to withdraw from the study at any time.

The researcher was a master’s degree candidate in psychological nursing and a psychological consultant in hospitals, and had received suicide-related communication and interview skills training and specialized in the suicide and psychology of patients for more than 1 year. During the study, the researcher provided psychological support to patients and referred them to the psychology or psychiatry department when necessary. Additionally, once patients with suicidal ideation were identified, related medical staff were requested to closely monitor the patients.

#### Statistical analysis

The questionnaires were independently input by the researchers, and data were sorted. The chi-squared test was used for univariate analysis using SPSS 22.0. Logistic regression analysis was conducted to screen for independent factors associated with suicidal ideation in lung cancer patients, and variables with *p* < 0.1 in univariate analysis were included in the regression analysis.

### Part 2: Qualitative component of the study

#### Design and participants

In this qualitative study, a phenomenological approach was adopted because it provides an opportunity to understand the meaning of a phenomenon (15). In the field of nursing, phenomenological methods are mainly used to explore subjective cognitive aspects or life experiences related to health and disease (16). This method was considered most relevant to this study as it allowed participants to focus on their personal feelings and describe the reasons for their suicidal ideation. It attempted to provide a better understanding and present their subjective perceptions in a clear and meaningful way. In Part 1, patients with lung cancer and suicidal ideation were further selected as interview subjects. The specific inclusion criteria were as follows: (a) age above 18 years; (b) >0 score in Item 9 of PHQ-9; (c) capacity to be independently conscious and understand the contents of the study; and (d) selection of patients of different ages, marital status, occupation, characteristics of lung cancer, length of diagnosis, hospitalization times, and other aspects to meet the maximum differentiation of background information. Patients with language communication difficulties were excluded. The final sample size was determined based on the principle of data saturation, i.e., when repeated information appeared in the interview analysis and no new information appeared.

#### Data collection

In the early stage, the interview outline was preliminarily formulated based on the literature review and research purpose. Experts on suicide and psychological nursing, head nurses of the lung cancer department, and members of the research group were invited to conduct thematic discussions, and the interview outline was revised. Two patients with lung cancer and suicidal ideation were pre-interviewed to familiarize the researchers with the study process and improve the analysis of the study. Therefore, the outline of the final interview was revised (Table 1). The participants were interviewed based on the guidelines of a semi-structured interview draft.

**Table 1:** Semi-structured interview guide.

N	Questions
1	Can you talk about the view and understanding of lung cancer before and after the disease? How do you feel about the treatment and prognosis of your disease?
2	Would you please tell me something about your family? Such as your primary caregiver, family finance, family support, etc.
3	Do you think your lung cancer has any impact on your family or friends?
4	Could you talk about the physical and psychological effects of lung cancer on you? What’s bothering you? For example, are there any symptoms of illness, mood or negative events in your life? What is the thing that bothers you the most?
5	How do you think about suicide? Under what circumstances would you have the idea or action that is not as good as death? Do the things that are bothering you have anything to do with wanting to die?

Under the principle of patient convenience, face-to-face semi-structured interviews were conducted in a quiet and separate room. Prior to the formal interview, patients’ medical records were checked to understand their basic information, current symptoms, disease progression, and treatment. The purpose, method, content, and voluntary and confidential principles of the interview were explained to the patients to establish a trusting relationship with them. Questions were asked as naturally as possible, and the order and manner of asking questions were flexibly adjusted according to patients’ sex, age, personality, and educational level to make the process simpler for them. In order to obtain valuable answers, ask further questions according to the purpose of this study after the patient has fully expressed, for example, in which situation your suicidal ideation would occur or disappear. Each interview lasted approximately 30 to 60 min, and the entire interview process was recorded. After the interview, patients were asked to check whether the collected data were consistent with their wishes to improve accuracy.

#### Data analysis

Interview contents were recorded by combining audio recordings and notes. Two researchers independently transcribed all the content provided by patients in the interview into text data word for word within 24 h by repeatedly listening to the audio recording, combining it with on-site notes, and anonymously coding each piece of data. When the results were inconsistent, a discussion was conducted by a third investigator. The analysis of qualitative data was based on the principle of researchers’ introspection and comparison and was completed through researchers’ thinking and induction. By sorting out and concluding qualitative data, researchers extract the theme.In the analysis, all criticism, comments, or opinions were avoided, and data were analyzed repeatedly to determine the nature of the interview content.. This study adopted Colaizzi’s seven-step data analysis method (17). To avoid subjective bias in the analyzed data, the transcribed text was returned to the patients for confirmation after data analysis to ensure the authenticity and accuracy of the results. In the case of inconsistencies, the research group discussed and determined the topic.

#### Ethical considerations

Ethical approval was obtained from the Institutional Ethics Review Board (approval number S171). The researchers strictly adhered to the standard ethical guidelines. As part of the informed consent, the participants were informed about the purpose and method of the study, the voluntary nature and anonymity of participants, and the right to withdraw at any time.

## Results

### Sociodemographic and clinical characteristics of lung cancer patients

Of the 366 patients with lung cancer that were investigated, 257 (70.2%) were men and 109 (29.8%) were women. The ages of the participants ranged from 19 to 88 years; 315 were married, four were unmarried, 12 were divorced, and 35 were widowed. There were 206 cases of lung adenocarcinoma, 93 cases of lung squamous cell carcinoma, and 67 cases of small cell lung cancer. In addition, there were 20, 70, 155, and 121 cases of stage I, II, III, and IV lung cancer, respectively. Table 2 shows the sociodemographic and clinical characteristics of the inpatients with lung cancer.

**Table 2:** Sociodemographic and clinical characteristics of lung cancer inpatients, with and without suicidal ideation.

Variable	Total N (%), *N* = 366	Patients with SI *N* (%), *N* = 83	Patients without SI *N* (%), *N* = 283	*X^2^*	*p*
Sex				9.483	0.002**
Male	257 (70.2)	47 (56.6)	210 (74.2)		
Female	109 (29.8)	36 (43.4)	73 (25.8)		
Age				7.816	0.100
18–29	3 (0.8)	2 (2.4)	1 (0.4)		
30–39	6 (1.6)	1 (1.2)	5 (1.8)		
40–49	34 (9.3)	6 (7.2)	28 (9.9)		
50–59	105 (28.7)	31 (37.3)	74 (26.1)		
≥60	218 (59.6)	43 (51.9)	175 (61.8)		
Employment status				0.747	0.387
Employed	101 (27.6)	26 (31.3)	75 (26.5)		
Unemployed	265 (72.4)	57 (68.7)	208 (83.5)		
Marital status				2.031	0.566
Non-married	4 (1.0)	2 (2.4)	2 (0.7)		
Married	315 (86.1)	69 (83.2)	246 (86.9)		
Divorced	12 (3.3)	3 (3.6)	9 (3.2)		
Death of a spouse	35 (9.6)	9 (10.8)	26 (9.2)		
Place of residence				0.993	0.319
Rural area	133 (36.3)	34 (41.0)	99 (35.0)		
Urban area	233 (63.7)	49 (59.0)	184 (64.0)		
Educational attainment				4.367	0.224
Primary school or lower	99 (27.0)	28 (33.7)	71 (25.1)		
Junior high school	160 (43.7)	32 (38.6)	128 (45.2)		
Senior high school	87 (23.8)	21 (25.3)	66 (23.3)		
University or higher	20 (5.5)	2 (2.4)	18 (6.4)		
Religion				4.269	0.039**
No	352 (96.2)	83 (100.0)	269 (95.1)		
Yes	14 (3.8)	0 (0.0)	14 (4.9)		
Monthly average household income (RMB)				5.645	0.227
<2,000	82 (22.4)	22 (26.5)	60 (21.2)		
2,000-3,000	42 (11.5)	8 (9.6)	34 (12.1)		
3,000-4,000	48 (13.1)	12 (14.5)	36 (12.7)		
4,000-5,000	43 (11.7)	14 (16.9)	29 (10.2)		
>5,000	151 (41.3)	27 (32.5)	124 (43.8)		
Smoking history(years)				18.257	0.000**
No-smoking	153 (41.8)	48 (57.8)	105 (37.1)		
1–20	53 (14.5)	4 (4.8)	49 (17.3)		
20–40	122 (33.3)	28 (33.8)	94 (33.2)		
>40	38 (10.4)	3 (3.6)	35 (12.4)		
Lung cancer types				0.130	0.937
Squamous cell lung carcinoma	93 (25.4)	20 (24.1)	73 (25.8)		
Adenocarcinoma of lung	206 (56.3)	47 (56.6)	159 (56.2)		
Small cell lung cancer	67 (18.3)	16 (19.3)	51 (18.0)		
Cancer stage				21.130	0.000**
I	21 (5.7)	0 (0.0)	21 (7.4)		
II	71 (19.4)	15 (18.1)	56 (19.8)		
III	155 (42.3)	25 (30.1)	130 (45.9)		
IV	119 (32.6)	43 (51.8)	76 (26.9)		
Months since cancer diagnosis				12.490	0.029**
1-3	111 (30.4)	19 (22.9)	92 (32.6)		
4–6	81 (22.1)	13 (15.7)	68 (24.0)		
7–9	51 (13.9)	14 (16.9)	37 (13.1)		
10–12	36 (9.8)	15 (18.1)	21 (7.4)		
13–24	57 (15.6)	15 (18.1)	42 (14.8)		
>24	30 (8.2)	7 (8.3)	23 (8.1)		
Hospitalized times				11.478	0.009**
First time	74 (20.2)	11 (13.3)	63 (22.3)		
2–3	84 (23.0)	23 (27.7)	61 (21.5)		
4–9	132 (36.1)	23 (27.7)	109 (38.5)		
>10	76 (20.7)	26 (31.3)	50 (17.7)		
Number of uncomfortable symptoms				27.204	0.000**
No symptoms	43 (11.7)	3 (3.6)	40 (14.1)		
1–2	228 (62.3)	41 (49.4)	187 (66.1)		
≥3	95 (26.0)	39 (47.0)	56 (19.8)		
Satisfaction of treatment				36.277	0.000**
Not satisfied	58 (15.8)	30 (36.1)	28 (9.9)		
Satisfied	190 (52.0)	39 (47.0)	151 (53.4)		
Very satisfied	118 (32.2)	14 (16.9)	104 (36.7)		

The above variables are categorical variables. All *p* values were estimated using a chi-square test. *X*^2^ chi-square test value. SI, suicidal ideation. **p<0.05.

In this study, 83 (22.68%) of the 366 patients with lung cancer reported suicidal ideation based on the score in Item 9 of PHQ-9. Results from the univariate analysis showed statistically significant differences among patients with lung cancer, with and without suicidal ideation, in the following aspects: sex, religion, smoking history, cancer stage, months since cancer diagnosis, number of hospitalizations, major discomfort symptoms, and satisfaction with the treatment (*p* < 0.05) (Table 2).

Significant variables from the univariate analysis were included in the logistic regression. Results showed that sex, cancer stage, number of uncomfortable symptoms, and satisfaction with treatment were independently associated with suicidal ideation in lung cancer patients. The incidence of suicidal ideation was higher in women, patients with advanced lung cancer, those with more symptoms, and those who were unsatisfied with the treatment (Table 3).

**Table 3:** Logistic regression analysis of factors independently associated with suicidal ideation in lung cancer patients.

Variable	*β*	SB	Wald	*P*	OR	95% CI
Sex	0.344	0.131	6.893	0.009	1.410	1.091 ~ 1.823
Cancer stage	0.418	0.150	7.735	0.005	1.519	1.131 ~ 2.038
Number of uncomfortable symptoms	0.512	0.153	11.184	0.001	1.668	1.236 ~ 2.251
Satisfaction with treatment	−0.633	0.146	18.895	<0.001	0.531	0.399 ~ 0.706
Constant term	−1.525	0.157	94.883	<0.001	0.218	—

### Influencing factors of suicidal ideation in lung cancer patients

Based on data saturation, eight hospitalized patients with lung cancer and suicidal ideation were selected as interviewees (Table 4). Analysis of these patients revealed three main themes: physiological, psychological, and social factors. Table 5 presents patients’ specific statements.

**Table 4:** Interviewees’ general information (*n* = 8).

N	Sex	Age	Marital status	Occupation	Lung cancer type	Cancer stage	Months since cancer diagnosis	Number of times hospitalized	Transfer or not
P1	Male	61	Married	Principal	Adenocarcinoma of lung	IV	48	>20	Yes
P2	Male	58	Married	Painter	Adenocarcinoma of lung	III	6	2	No
P3	Male	63	Divorced	Sector	Squamous cell carcinoma of lung	III	30	>3	Yes
P4	Female	67	Married	Farmer	Squamous cell carcinoma of lung	IV	30	>40	Yes
P5	Male	32	Non-married	Unemployed	Adenocarcinoma of lung	III	4	2	Yes
P6	Female	59	Married	Retire	Squamous cell carcinoma of lung	II	1	2	Yes
P7	Male	52	Married	Pharmacist	Adeno-squamous carcinoma of lung	IV	12	8	Yes
P8	Female	68	Married	Farmer	Small cell lung cancer	IV	12	>10	Yes

**Table 5:** Themes extraction of interview data from hospitalized lung cancer patients with suicidal ideation.

Main themes	Sub-themes	Raw data
1. Physiological factors	1.1 Heavy symptom burden	P3: “*I cannot walk now. Half of my sternum hurts. I cannot even take my clothes off. I think about it all the time in my family. It’s painful. It’s too painful. I was in pain and pain at home (cried)…”*	P4: *“I’ve been hospitalized more than 40 times. I cannot stand it and cannot take care of myself. Now I have pericardial effusion, pleural effusion, vomiting, and coughing. I also cannot sleep. Suicidal ideation occurs when the body is particularly uncomfortable and cannot stand it, and also when the body cannot move…”*
2. Psychological factors	2.1 Bad mood	P1: “*I was so anxious that I would wake up five to six times every night. I even think that the treatment of lung cancer is ineffective. I do not want to treat it, it is useless to treat it, and the money is spent badly. I have no hope for the recovery of my body. Over and over, the pain in my heart is more than the pain in my body, the heart is bitter(cried)…”*	P8: “*I have been desperate, do not want to treat, It hurts! the effect is not good, I hurt more than a month, also do not have a very good solution, I spend every day in pain…”*	2.2 Thwarted belongingness	P3: “*It’s impossible for my daughter to take care of me. I was divorced, and my wife died of asthma, but I took care of everything, but now I’m sick and nobody cares(cried). My daughter has not been here since my illness, not even to call me, I hate her very much…”*	P5: “*I used to have a girlfriend, but now it’s over. Now I spend a lot of time in the hospital, and I cannot play with my friends. I do not have much contact with them, so my friendship may weaken a lot…”*	2.3 Perceived burdensomeness	P6: “*My illness has affected my children’s work and their moods. They have to worry about their work as well as me. There is no one to manage the grandson now, now the grandson has to sent to nursery class…”*	P8: “*I spent a lot of money on my medical treatment. I’m a burden on my family. I told my husband, you let me die, and you will be fine, because I also owe money outside, and they are spending all money on me. I’ve spent all the money. What are they going to do? Pick up trash?…”*	2.4 Stigma	P1: “*After my illness, my friends stayed away from me and were reluctant to contact me. Everyone looked at me differently. And everyone in my area knew I had cancer, it was like a guilty conscience…”*	P2: *“Some others discriminate against me, say lung cancer can infect, do not contact him. I stopped being in their circle, did not want to meet them, and slowly left the group. It’s best to be alone now and do not want to associate with outsiders…”*
3. Social factors	3.1 High economic pressure	P5: “*Now the family depends on my father, he is only a worker, the family economic pressure is very big. Borrowed some money from relatives at home for treatment. I ran out of money and could not get reimbursed for his treatment…”*	P6: “*My husband has to take care of me, and not making any money now, not a cent. I now regret, if I knew to spend so much money, I would rather die than come to the treatment. There is no money at home, I have been crying and crying, anyway, the cure is not good. After I get the disease, I have the idea that I do not want to live. I know my condition, and I have no money to treat it. I also know that this disease is not long to live…”*	3.2 Negative life events	P3: “*After my divorce, my daughter followed her mother, who was dead, and she lost money in her own business. My sister had breast cancer too…”*	P7: “*My wife is here to take care of me. She had ovarian cancer before…”*

### Theme 1: Physiological factors (heavy symptom burden)

Since the early symptoms of lung cancer are relatively insidious and lack specificity, most patients are already in the advanced stage when they seek treatment. The heavy burden of disease symptoms in patients with advanced disease greatly affects their quality of life. Patients can no longer tolerate their life and gradually lose confidence in treatment, leading to a sense of despair and the idea of dying.

### Theme 2: Psychological factors

#### Bad mood

The inability to cope with the disease, poor treatment effect, and unbearable costs of treatment cause patients to lose heart. When patients lose confidence and hope of changing their current state, they feel extremely low and experience psychological pain, which leads to a sense of despair. In this interview, despair and psychological pain were common in the patients’ descriptions.

#### Thwarted belongingness

In addition, the thwarted belongingness in patients with lung cancer was manifested as loneliness induced by the lack of family care and company after the disease. Particularly in patients with no familial care, these manifestations included a lack of support from family, a belief that they were abandoned even in the late stages of cancer, the thought that no one cared, an inability to obtain help or moral support from others, frustration from negative performance, loneliness, and a failure to appropriate emotional outpouring and support.

#### Perceived burdensomeness

Patients in hospitals need familial care. On the one hand, their daily care requires a great amount of effort from the family, which can be a great burden on the entire family. On the other hand, families may give up their jobs or make arrangements to take care of their patients. In addition, the treatment of disease imposes a serious financial burden on the family. Therefore, patients are prone to a fatal illusion of “if they die, the family will be relaxed and happy,” which leads to suicidal ideation.

#### Stigma

As the symptoms of lung cancer are similar to those of infectious diseases, such as tuberculosis, people may think that lung cancer is contagious and may have prejudice against patients. In addition, people would equate lung cancer with “death.” Influenced by traditional culture, people are sensitive about “death”; therefore, they avoid patients. Such discriminatory experiences lead to inner shame in patients.

### Theme 3: Social factors

#### High economic pressure

The cost of lung cancer diagnosis and treatment can be devastating for patients and families, imposing a severe financial burden. Patients with lung cancer are repeatedly hospitalized for a long time, which means that hospitalization costs constantly increase. The cost is difficult to bear even for families with better circumstances; for ordinary or poor rural families, it is undoubtedly a bolt from the blue. Thus, most patients with lung cancer are waiting for their deaths.

#### Negative life events

Before and after patients are diagnosed with the disease, they may be exposed to negative life events, such as the death of a family member, serious illness in the family, work setbacks, and family tensions. In addition, long-term emotional isolation can extremely harm their health, coupled with the diagnosis of lung cancer. When sad emotionally isolated patients can not properly vent negative emotions, such as long-term guilt, more serious psychological problems occur.

## Discussion

This study highlights the importance of paying attention to suicidal ideation in lung cancer patients. It has found that the incidence of suicidal ideation in lung cancer patients is 22.68%, which is higher than an earlier survey of Chinese community groups. (2.20%) (18) and in patients with stomach cancer and gynecological malignant tumors (10, 11). In addition, Vyssoki et al. (6) has found that lung cancer patients have a higher incidence of suicidal ideation than those with other cancers (6). This proves that, in China, lung cancer patients are at a higher risk of suicide than those with other cancers, which is consistent with foreign studies. Furthermore, patients have specific risk factors for suicidal ideation. Therefore, it is important that medical staff understand the influencing factors of suicidal ideation. Patients should be closely monitored for suicidal ideation and provided with specialized care to reduce the risk of suicide in the short and long term (19).

Logistical regression analysis showed that sex (OR = 1.410, 95% CI: 1.091–1.823), cancer stage (OR = 1.519, 95% CI: 1.131–2.038), number of uncomfortable symptoms (OR = 1.668, 95% CI: 1.236–2.251), and satisfaction with treatment (OR = 0.531, 95% CI: 0.399–0.706) were independent risk factors for suicidal ideation in lung cancer patients. A survey in South Korea showed that lung cancer with a malignant tumor is associated with the highest suicide risk among women (20). Another study revealed that female lung cancer patients were more likely to report suicidal ideation than male lung cancer patients, which was a similar finding to the results of this study (21). This may be related to the fact that, compared with male patients, female patients are more prone to stress resistance and mental toughness, severe depression, and other negative affectivities. Therefore, female patients were more likely to have suicidal ideation than male patients (22). The results of this study showed that the later the stage of lung cancer, the higher the incidence of suicidal ideation. This was consistent with the findings of related studies on the risk of suicide in lung cancer patients (23, 24), and the long-term and unpredictable course of the disease has led to a surge in suicide cases in advanced lung cancer patients (4). Advanced lung cancer patients suffer more because of body symptoms and their inability to self-care and control their daily life; they often had severe anxiety, depression, and other negative emotional experiences, resulting in a decrease in their quality of life, loss of confidence, and desperation. Therefore, such events lead to suicidal ideation (25).

In addition, this study indicates that the more uncomfortable the symptoms, the higher the incidence of suicidal ideation. A national multicenter study in Germany identified various symptoms in lung cancer patients; more severe symptoms of functional overlay resulted in patients experiencing unbearable pain, making them unable to provide for themselves (26). This eventually results in a reduced quality of life and, in turn, induces suicidal ideation. Additionally, the results of this study showed that treatment satisfaction was a protective factor for suicidal ideation. Treatment satisfaction means that patients can maintain an optimistic attitude and calmly tackle the disease. Furthermore, patients with mild disease and who positively respond to treatment have hope and confidence in their recovery; thus, they do not consider suicide an option.

Qualitative studies found that heavy symptom burden, bad mood, thwarted belongingness, perceived burdensomeness, stigma, high economic pressure, and negative life events might be closely related to suicidal ideation in Chinese patients diagnosed with lung cancer, which complemented the results of the quantitative study. The results have also shown the important influencing factors of suicide during the COVID-19 pandemic (27). Studies have indicated that compared with other malignant tumors, lung cancer is more refractory and progresses rapidly, with a severe burden of disease symptoms, including dyspnea, asthma, and fatigue (26, 28). These severe symptoms lead to the loss of the patient’s ability to take care of themselves. As patients suffer from repeated pain, they cannot bear it and want to end their lives. Moreover, lung cancer patients who reported suicidal ideation during the interview were more likely to experience severe cancer pain. In this study, several patients reported that they “sometimes felt so much pain and wanted to die.” Cases of suicide caused by cancer pain have often been reported (29, 30). Therefore, symptomatic treatment and standardized management of cancer pain are especially important.

The diagnosis of lung cancer involves intense physical and mental stimulations, resulting in a strong psychological stress response in patients. As the disease progressed and symptoms worsened, patients experienced a severe bad mood. In the interviews, patients with suicidal ideation had advanced lung cancer; therefore, dysthymia was more serious, including psychological pain and hopelessness. Moreover, the three-step theory of suicide has indicated that psychological pain and hopelessness are key factors in suicidal ideation (31). Therefore, medical personnel should routinely screen for psychological problems and focus on the psychological pain and hopelessness in patients, as well as early detection and timely intervention to promote the physical and mental health of patients. For patients with mental illness, such as depression, proper treatment measures should be taken (32). In addition, the sense of thwarted belongingness mainly manifested as a strong sense of loneliness (33). Studies have found that loneliness is common in lung cancer patients and is closely associated with suicidal outcomes, particularly suicidal ideation (34). Owing to the serious symptoms of lung cancer, complex treatment, and the change in living environment caused by hospitalization, patients gradually alienate from society. Moreover, their scope of communication narrows and they become lonely easily when their need for belongingness cannot be met. They feel abandoned by others, which leads to suicidal ideation. Therefore, it is necessary to focus on the patient’s social support system, particularly family support, to accompany and care for the patient and increase their sense of belongingness.

In addition, owing to patients’ illnesses and the care they need, they tend to feel guilty about their overdependence on family members and the burden this places upon them (35). Zhang Hui had shown that up to 98.2% of patients had perceived burdensomeness, among which 67.0% had moderate-to-severe lung cancer (36). Patients with perceived burdensomeness often felt that they could not contribute to others, that their presence was a nuisance, and that if they died, others would be better off (37). Perceived burdensomeness can be reduced by affirming patients’ contribution to the family and society and increasing their sense of value. In addition, stigma is considered a potential risk factor for suicidal ideation (38). Studies have found that stigma is common in lung cancer patients, and its level is higher than that in other cancer patients (39, 40). As smoking is closely associated with lung cancer, patients feel that it is a self-inflicted behavior, and the clinical symptoms of lung cancer are similar to those of some infectious diseases. Individuals may avoid patients because of the fear of being infected, which leads to the stigma of patients being discriminated against. Therefore, the stigma of lung cancer patients should be reduced through health education, personalized alternative methods, and cognitive behavioral therapy to prevent further deterioration of patients’ psychological well-being.

Lung cancer treatment can lead to devastating medical costs for families, particularly those in low-and middle-income families. Lung cancer patients have a heavy financial burden, with an average annual burden of nearly 126,100 RNB, based on a survey in China (41). Additionally, a study in Iran indicated that the economic burden of lung cancer treatment had a huge impact on the country’s health system and society as a whole (42). In rural areas of China in particular, family income is low and coupled with low rates of medical insurance reimbursement, resulting in great economic pressure. This greatly aggravates the burden on patients, who believe that the family would be more relaxed and happier if they died. In the interview, some lung cancer patients also suffered from negative life events, such as the death of family members and illness. Numerous studies have confirmed that negative life events are closely associated with suicide, and most individuals who commit suicide have experienced negative life events in the past year (43). Therefore, medical staff should focus on patients who have had recent major accidents and provide timely psychological counseling and comfort to prevent suicides (44).

This study has several limitations. Owing to the small sample size and purposive sampling, lung cancer patients were recruited from one tertiary class hospital in Wuhan; therefore, this study may not be a representative of lung cancer patients in mainland China. In future, a large sample size and multicenter survey should be conducted in various regions of China. In addition, because of their culture and tradition, Chinese people are sensitive to words such as “death” or “suicide.” In this study, only the last item of PHQ-9 was used to measure suicidal ideation, instead of using the professional assessment scale. Nevertheless, Item 9 of PHQ-9 has good psychometric characteristics, is considered a reliable tool, and is easily accepted by Chinese lung cancer patients. Finally, this qualitative study did not determine when patients have suicidal ideation and when this ideation disappears. In the future, more in-depth interviews should be conducted to determine the course of suicidal ideation. Nonetheless, to the best of our knowledge, this is the first study to describe the incidence and factors influencing suicidal ideation in lung cancer patients. It not only adopted a cross-sectional survey to analyze the relevant demographic and disease characteristics but also used qualitative interviews to obtain an in-depth understanding of the causes of suicidal ideation in lung cancer patients.

## Conclusion

Evidently, the incidence of suicidal ideation in lung cancer patients is high, which is a significant global health problem. The results showed that the incidence of suicidal ideation in lung cancer patients was 22.68%; women, advanced lung cancer patients, those who had severe symptoms of discomfort, and those who were not satisfied with the treatment had a higher incidence. This study also revealed three major themes among patients with suicidal ideation who were diagnosed with lung cancer: physiological (heavy burden of symptoms), psychological (bad mood, thwarted belongingness, perceived burdensomeness, and stigma), and social factors (high economic pressure and negative life events). Each factor suggests a risk for suicidal ideation and can play an important role in future suicide prevention programs for lung cancer patients. Nursing practitioners should focus on screening and intervention in lung cancer patients at high risk of suicidal behaviors.

## Data availability statement

The original contributions presented in the study are included in the article/supplementary material, further inquiries can be directed to the corresponding author.

## Ethics statement

The studies involving human participants were reviewed and approved by Ethics Committee of Huazhong University of Science and Technology. The patients/participants provided their written informed consent to participate in this study.

## Author contributions

TY, DYH, and YJ: conception and design. TY and CW: provision of study materials. TY and SSL: collection and assembly of data. TY, DYH, YJ, CW, and SSL: manuscript writing. All authors contributed to the article and approved the submitted version.

## Funding

This study was funded by the National Natural Science Foundation of China (71673100).

## Conflict of interest

The authors declare that the research was conducted in the absence of any commercial or financial relationships that could be construed as a potential conflict of interest.

## Publisher’s note

All claims expressed in this article are solely those of the authors and do not necessarily represent those of their affiliated organizations, or those of the publisher, the editors and the reviewers. Any product that may be evaluated in this article, or claim that may be made by its manufacturer, is not guaranteed or endorsed by the publisher.

## References

[1] World Health Organization. (2018). Estimated number of new cases in 2018, worldwide, both sexes, all ages. Available at: https://www.coursehero.com/file/p6bn284i/Estimated-number-of-new-cases-in-2018-all-cancers-both-sexes-all-ages-China-4/(Accessed October 6, 2022).

[2] Odone A Lugo A Amerio A Borroni E Bosetti C Carreras G. COVID-19 lockdown impact on lifestyle habits of Italian adults. Acta Biomed. (2020) 91:87–9. doi: 10.23750/abm.v91i9-S.10122 32701921 PMC8023096

[3] Khue PM Thom VT Minh DQ Quang LM NL H. Depression and anxiety as key factors associated with quality of life among lung cancer patients in Hai Phong, Vietnam. Front Psychiatry. (2019) 10:352. doi: 10.1038/s41415-019-0440-2, PMID: 31156487 PMC6532361

[4] Rahouma M Kamel M Abouarab A Eldessouki I Nasar A Harrison S. Lung cancer patients have the highest malignancy-associated suicide rate in USA: a population-based analysis. E Cancer Med Sci. (2018) 12:859. doi: 10.3332/ecancer.2018.859, PMID: 30174721 PMC6113987

[5] Klaassen Z Wallis C Chandrasekar T Goldberg H Sayyid RK Williams SB. Cancer diagnosis and risk of suicide after accounting for prediagnosis psychiatric care: a matched-cohort study of patients with incident solid-organ malignancies. Cancer. (2019) 125:2886–95. doi: 10.1002/cncr.32146, PMID: 31021430 PMC10182898

[6] Vyssoki B Gleiss A Rockett IR Hackl M Leitner B Sonneck G. Suicide among 915,303 Austrian cancer patients: who is at risk? J Affect Disord. (2015) 175:287–91. doi: 10.1016/j.jad.2015.01.028, PMID: 25661393

[7] The United Nations. (2020). International Agency for Research on Cancer: 19.3 million new cancer patients have added to the world in 2020, 10 million dead of cancer. Available at: https://news.un.org/zh/story/2020/12/1073672(Accessed October 6, 2022).

[8] Suicide Prevention Resource Center. (2014). Suicide screening and assessment. Available at: http://www.sprc.org/sites/default/files/migrate/library/RS_suicide%20screening_91814%20final.pdf(Accessed October 6, 2022).

[9] Baca-Garcia E Perez-Rodriguez MM Oquendo MA Keyes KM Hasin DS Grant BF. Estimating risk for suicide attempt: are we asking the right questions? Passive suicidal ideation as a marker for suicidal behavior. J Affect Disord. (2011) 134:327–32. doi: 10.1016/j.jad.2011.06.026, PMID: 21784532 PMC3172880

[10] Tang GX Yan PP Yan CL Fu B Zhu SJ Zhou LQ. Determinants of suicidal ideation in gynecological cancer patients. Psycho-Oncology. (2016) 25:97–103. doi: 10.1002/pon.3880, PMID: 26103593

[11] Xue-kun Z. Suicidal Ideation in Patients with Stomach Cancer: Relationship with Psychological Strain and Intervention Development. Jinan: Shang Dong University (2018).

[12] Zheng-Qiu S. Medical statistics. 3rd ed. Beijing: People's Medical Publishing House (2010).

[13] Kroenke K Spitzer RL Williams JB. The PHQ-9: validity of a brief depression severity measure. J Gen Intern Med. (2001) 16:606–13. doi: 10.1046/j.1525-1497.2001.016009606.x, PMID: 11556941 PMC1495268

[14] Ke H De-ying H Rong T Xiao-ping D Fen T Yi Z. Research progress on patient suicide risk screening and assessment. Chin J Nurs. (2019) 54:467–71. doi: 10.3761/j.issn.0254-1769.2019.03.029

[15] Barnett M. Chronic obstructive pulmonary disease: a phenomenological study of patients' experiences. J Clin Nurs. (2005) 14:805–12. doi: 10.1111/j.1365-2702.2005.01125.x 16000094

[16] Refrande SM Silva R Pereira ER Rocha R Melo S Refrande NA. Nurses' experiences in the care of high-risk newborns: a phenomenological study. Rev Bras Enferm. (2019) 72:111–7. doi: 10.1590/0034-7167-2018-0221, PMID: 31851242

[17] Ming L. Using an example to illustrate Colaizzi’S phenomenological data analysis method. J Nurs Sci. (2019) 34:90–2. doi: 10.3870/j.issn.1001-4152.2019.11.090

[18] Ma X Xiang YT Cai ZJ Li SR Xiang YQ Guo HL. Lifetime prevalence of suicidal ideation, suicide plans and attempts in rural and urban regions of Beijing, China. Aust N Z J Psychiatry. (2009) 43:158–66. doi: 10.1080/00048670802607170, PMID: 19153924

[19] Heinrich M Hofmann L Baurecht H Kreuzer PM Knuttel H Leitzmann MF. Suicide risk and mortality among patients with cancer. Nat Med. (2022) 28:852–9. doi: 10.1038/s41591-022-01745-y 35347279

[20] Ahn E Shin DW Cho SI Park S Won YJ Yun YH. Suicide rates and risk factors among Korean cancer patients, 1993-2005. Cancer Epidemiol Biomark Prev. (2010) 19:2097–105. doi: 10.1158/1055-9965.EPI-10-0261, PMID: 20696665

[21] Kye SY Park K. Suicidal ideation and suicidal attempts among adults with chronic diseases: a cross-sectional study. Compr Psychiatry. (2017) 73:160–7. doi: 10.1016/j.comppsych.2016.12.001, PMID: 27992846

[22] Boyd A Van de Velde S Vilagut G de Graaf R O'Neill S Florescu S. Gender differences in mental disorders and suicidality in Europe: results from a large cross-sectional population-based study. J Affect Disord. (2015) 173:245–54. doi: 10.1016/j.jad.2014.11.002, PMID: 25462424

[23] Kaceniene A Danila E Cicenas S Smailyte G. Suicide risk among lung cancer patients in Lithuania. Clini Respirat J. (2018) 12:2455–6. doi: 10.1111/crj.12916, PMID: 30073786

[24] Stephanie M Weiss NS Fann JR Mary R Bevan Y. Incidence of suicide in persons with cancer. J Clin Oncol. (2008) 26:4731–8. doi: 10.1200/JCO.2007.13.8941 18695257 PMC2653137

[25] Park SA Chung SH Lee Y. Factors associated with suicide risk in advanced cancer patients: a cross-sectional study. Asian Pac J Cancer Prev. (2016) 17:4831–6. doi: 10.22034/APJCP.2016.17.11.4831 PMID: 28030907 PMC5454682

[26] Kuon J Vogt J Mehnert A Alt-Epping B van Oorschot B Sistermanns J. Symptoms and needs of patients with advanced lung cancer: early prevalence assessment. Oncol Res Treat. (2019) 42:650–9. doi: 10.1159/000502751, PMID: 31634889

[27] Farooq S Tunmore J Wajid AM Ayub M. Suicide, self-harm and suicidal ideation during COVID-19: a systematic review. Psychiatry Res. (2021) 306:114228. doi: 10.1016/j.psychres.2021.114228, PMID: 34670162 PMC8495045

[28] Weller D. "yes, I have cancer, but I'm also lonely"; tackling a common problem in cancer care. Eur J Cancer Care. (2018) 27:e12844. doi: 10.1111/ecc.12844, PMID: 29575266

[29] Yue-mei C，Yuan-ping P，Lin T，Guo-bi W, Yan-yan W, Cui-e G. Application of root cause analysis in the suicide management of hospitalized cancer patients. J Nurs (2014), 21: 13–14. doi: 10.16460/j.issn1008-9969.2014.18.010

[30] Fishbain DA. Predictive factors for suicidal ideation in patients with unresectable lung carcinoma. A 6-month follow-up study. Cancer. (2003) 97:3127–3128, 3129. doi: 10.1002/cncr.11429, PMID: 12784353

[31] Klonsky ED May AM. The three-step theory (3ST): a new theory of suicide rooted in the “ideation-to-action” framework. Int J Cogn Ther. (2015) 23:648–61. doi: 10.1080/13811118.2018.1497563

[32] Belvederi MM Ekkekakis P Magagnoli M Zampogna D Cattedra S Capobianco L. Physical exercise in major depression: reducing the mortality gap while improving clinical outcomes. Front Psych. (2018) 9:762. doi: 10.3389/fpsyt.2018.00762, PMID: 30687141 PMC6335323

[33] Hyland KA Small BJ Gray JE Chiappori A Creelan BC Tanvetyanon T. Loneliness as a mediator of the relationship of social cognitive variables with depressive symptoms and quality of life in lung cancer patients beginning treatment. Psychooncology. (2019) 28:1234–42. doi: 10.1002/pon.5072, PMID: 30932275

[34] Calati R Ferrari C Brittner M Oasi O Olie E Carvalho AF. Suicidal thoughts and behaviors and social isolation: a narrative review of the literature. J Affect Disord. (2019) 245:653–67. doi: 10.1016/j.jad.2018.11.022, PMID: 30445391

[35] Miao X，Zhe S，Liu Y，Bin-bin W，Yu-li Y. The effect of self-perceived burden on the quality of life of lung cancer patients undergoing chemotherapy. Chin J Gerontol. (2020). 40: 880–883. doi: 10.3969/j.issn.1005-9202.2020.04.064

[36] Hui Z Gui-xia L. Correlation between self - perceived burden and disease perception, optimism and post - traumatic growth in patients with lung cancer during chemotherapy. Guangdong Med J. (2020) 41:169–73. doi: 10.13820/j.cnki.gdyx.20191680

[37] LeRoy AS Lu Q Zvolensky MJ Ramirez J Fagundes CP. Anxiety sensitivity moderates the painful effects of feeling burdensome to others. Cogn Behav Ther. (2018) 47:126–38. doi: 10.1080/16506073.2017.1357749, PMID: 28791887 PMC6049813

[38] Fen G，Yi D，Michael，Huan-zhong L，Jian-liang H，Jun X，. Reliability and validity of the Chinese version of the stigma scale for mental illness. Chin Ment Health J. (2010). 24: 343–346. doi: 10.3969/j.issn.1000-6729.2010.05.007

[39] Oexle N Rusch N. Stigma - risk factor and consequence of suicidal behavior: implications for suicide prevention. Nervenarzt. (2018) 89:779–83. doi: 10.1007/s00115-017-0450-8, PMID: 29147725

[40] Ostroff JS Riley KE Shen MJ Atkinson TM Williamson TJ Hamann HA. Lung cancer stigma and depression: validation of the lung cancer stigma inventory. Psychooncology. (2019) 28:1011–7. doi: 10.1002/pon.5033, PMID: 30779396 PMC6506363

[41] Xing-long W. Study on Economic Burden of Lung Cancer Patients and the Effect of Medical Insurance Policies in Nanchang. Jiangxi: Nan Chang University (2016).

[42] Rezaei S Akbari SA Woldemichael A Soofi M Kazemi A Karami MB. Estimating the economic burden of lung cancer in Iran. Asian Pac J Cancer Prev. (2016) 17:4729–33. doi: 10.22034/apjcp.2016.17.10.4729, PMID: 27893204 PMC5454624

[43] Cai-zhi W，Shuo R，Wen-ting D，Wei-xin W，Li-xia Y，. Effect of negative life events on suicide attempt: the mediating roles of basic psychological needs and Psychache. Chin J Clin Psych. (2020). 28: 503–507. doi: 10.16128/j.cnki.1005-3611.2020.03.015

[44] Nuij C van Ballegooijen W de Beurs D Juniar D Erlangsen A Portzky G. Safety planning-type interventions for suicide prevention: meta-analysis. Br J Psychiatry. (2021) 219:419–26. doi: 10.1192/bjp.2021.50, PMID: 35048835

